# An Electroencephalography-based Database for studying the Effects of Acoustic Therapies for Tinnitus Treatment

**DOI:** 10.1038/s41597-022-01622-w

**Published:** 2022-08-17

**Authors:** Alma Rosa Cuevas-Romero, Luz María Alonso-Valerdi, Luis Alejandro Intriago-Campos, David Isaac Ibarra-Zárate

**Affiliations:** 1 Tecnológico de Monterrey, Escuela de Ingeniería y Ciencias, Monterrey, N.L. México; 2 Universidad Autónoma de Nuevo León, Facultad de Ingeniería Mecánica y Eléctrica, San Nicolás de los Garza, N.L. México

**Keywords:** Quality of life, Prognostic markers

## Abstract

The present database provides demographic (age and sex), clinical (hearing loss and acoustic properties of tinnitus), psychometric (based on Tinnitus Handicapped Inventory and Hospital Anxiety and Depression Scale) and electroencephalographic information of 89 tinnitus sufferers who were semi-randomly treated for eight weeks with one of five acoustic therapies. These were (1) placebo (relaxing music), (2) tinnitus retraining therapy, (3) auditory discrimination therapy, (4) enriched acoustic environment, and (5) binaural beats therapy. Fourteen healthy volunteers who were exposed to relaxing music and followed the same experimental procedure as tinnitus sufferers were additionally included in the study (*control group*). The database is available at 10.17632/kj443jc4yc.1. Acoustic therapies were monitored one week after, three weeks after, five weeks after, and eight weeks after the acoustic therapy. This study was previously approved by the local Ethical Committee (CONBIOETICA19CEI00820130520), it was registered as a clinical trial (ISRCTN14553550) in BioMed Central (Springer Nature), the protocol was published in 2016, it attracted L’Oréal-UNESCO Organization as a sponsor, and six journal publications have resulted from the analysis of this database.

## Abstract (summary)

**Table:** 

Measurement(s)	brain electrical signals
Technology Type(s)	Electroencephalography
Factor Type(s)	EEG

## Background & Summary

Tinnitus is a multifactorial and heterogeneous condition highly associated with hearing loss (HL), age, sex, marital status, education, and even, employment. Furthermore, a large number of tinnitus sufferers (around 65%) eventually present sleep disorders, emotional and cognitive distress (e.g., stress, anxiety or depression) or any other mental condition^1^. In fact, around 70% of tinnitus cases are preceded by a mental disorder. Patients with highly distressing tinnitus can additionally have cardiovascular, endocrine or metabolic diseases^2^.

Tinnitus is a heterogeneous affection with no standard effective treatment. A recent study^3^ based on a survey conducted by the web platform Tinnitus Hub, where 5017 tinnitus sufferers participated, seven of the 25 most widely used treatment were sound based therapies. From the most to the least applied therapies, these were: 1^st^ self sound stimulation, 6^th^ masker, 11^th^ Tinnitus Retraining Therapy (TRT), 18^th^ notched music, 19^th^ soundcure, 20^th^ acoustic neuromodulation, and 21^st^ neuromonics. As can be seen, almost one third of the current available treatments for tinnitus depends on sound effect on body functioning. It is well-established that sound brings about physiological, cognitive and psychological changes^4^. However, it is still unknown how sound-based treatment alleviate or palliate tinnitus, and what is the appropriate therapy for each clinical case due to its heterogeneous nature.

To measure the sound effects of acoustic therapies on tinnitus, electroencephalographic (EEG) monitoring has been proposed. In^5^, authors presented a review of computational methods to analyse EEG signals for evaluating the sound effects of acoustic therapies at a cortical level. Over the past two years, the acoustic therapies that have been assessed are (1) neuromodulation, (2) auditory residual inhibition, (3) binaural beats therapy (BBT), and (4) auditive discrimination therapy (ADT). In the first case^6^, neuromodulation therapy was applied for 75 days, and EEG monitoring was undertaken every two weeks. It was found that the level of EEG connectivity diminished considerably. In the second case, auditory residual inhibition did not reduce significantly the tinnitus loudness, and this finding was supported by EEG evidence since band power in alpha and gamma increased in magnitude^7^. Similarly, alpha band power in auditory areas increased during auditory residual inhibition in the study conducted in^8^. In the third case, BBT reduced stress (23% of patients) and tinnitus perception (15% of patients), and it slightly reduced EEG synchronicity over the right frontal lobe^9^. In the last case, the level of EEG synchronicity due to auditory processing decreased due to the attentional redirection achieved by ADT^10^.

As can be seen from previous studies, EEG analysis is a helpful tool to evaluate the sound effects of acoustic therapies, in addition to psychoacoustic and clinical assessments. For this reason, the present database provides demographic, clinical, psychometric, and EEG information of 89 tinnitus sufferers who were randomly treated for eight weeks with one of five acoustic therapies. These were (1) relaxing music (placebo), (2) tinnitus retraining therapy (TRT), (3) ADT, (4) enriched acoustic environment (EAE), and (5) BBT. TRT seeks to reduce tinnitus by reducing the loudness perception of the unreal sound^11,12^. ADT intends to redirect the patient attention towards the therapy by presenting a composed sound of standard and deviant pulses in a random way, and thus reducing tinnitus perception^13^. EAE intends to prevent HL, and subsequent plastic tonotopic cortical map changes after acoustic trauma. EAE is based on a sequence of random frequency tones with amplitude proportional to the HL reported on the patient audiometry^14^. BBT consists of two pure tones presented on each ear, having a difference in frequency according to the target oscillatory EEG band. The use of this therapy has reported reduction of stress levels by reducing activation of areas in the sympathetic system^15^, what in turn reduce tinnitus perception. In addition to the 89 tinnitus sufferers, 14 healthy volunteers were recruited, exposed to relaxing music (same as control group), and followed the same experimental procedure, as tinnitus sufferers.

**Fig. 1:**
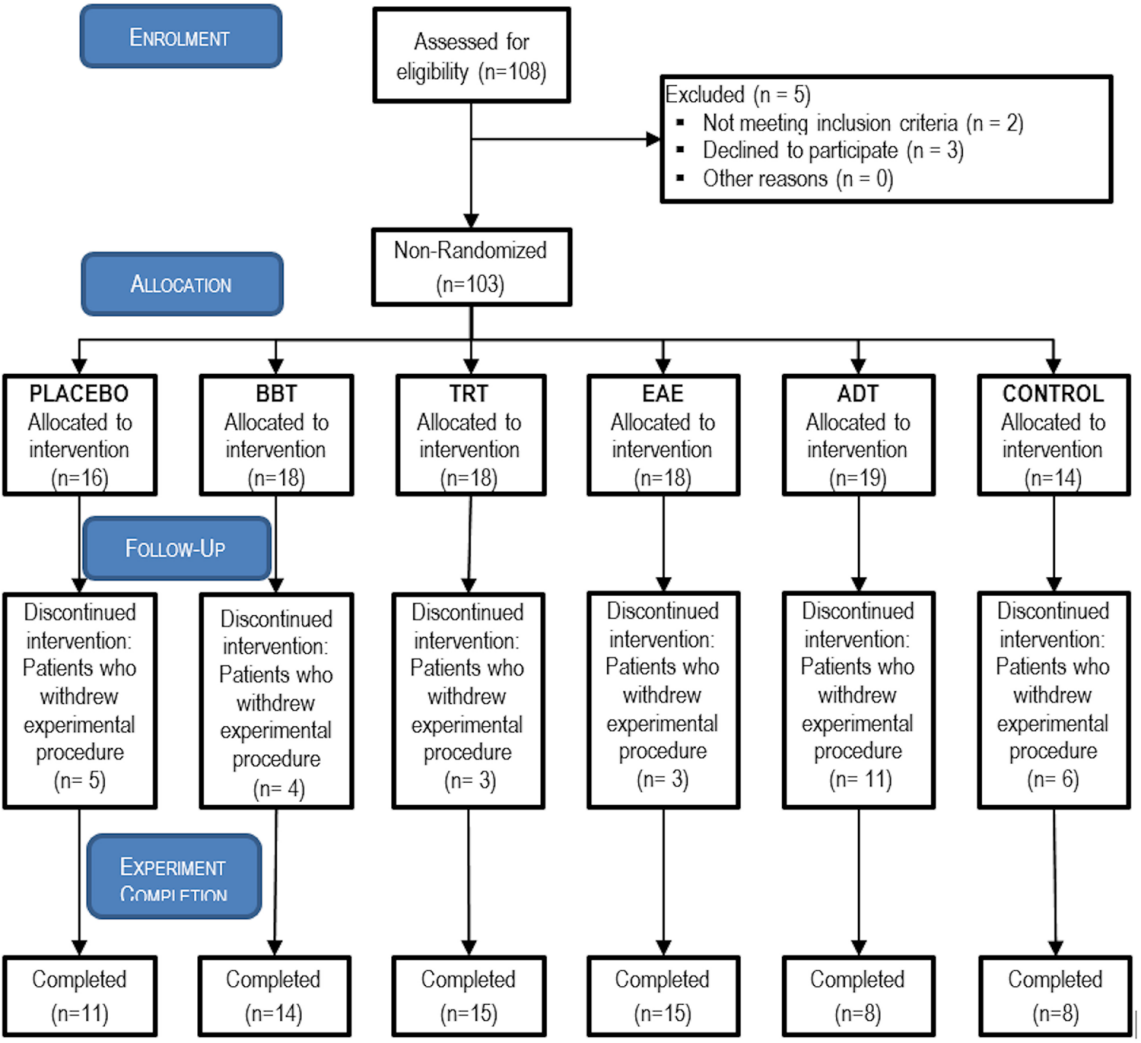
Flow diagram based on the Consolidated Standards of Reporting Trials. In total, 108 participants were recruited, 103 of them accepted to participate in the study, and 71 of them completed the experimental procedure.

In total, the database contains four types of information sources of 103 participants. See Table 1. First, d*emographic information* refers to residence place, gender, age (higher than 18-year-old), and nationality. Second, c*linical history* includes HL level, and frequency, laterality, and intensity of tinnitus perception. Finally, *psychometric and EEG monitoring* of the acoustic therapies is included, as well. It is important to note that the initial cohort was of 108 participants, 103 of them were included in the study, and 71 of them completed the experimental procedure. See Fig. 1.

**Table 1:** Total number of participants and their corresponding study group.

Group Number	Participant Condition	Number of Participants	Acoustic Therapy
1	Patient with tinnitus	16	Placebo
2		18	BBT
3		18	TRT
4		18	EAE
5		19	ADT
6	Healthy volunteer	14	Control
**TOTAL:**		**103**	
**MEAN:**		**17** ± **1.83**

*Psychometric monitoring* was based on the Spanish version of the Hospital Anxiety and Depression Stress (HADS), and the Tinnitus Handicap Inventory (THI), which provided psychoacoustic effect of acoustic therapies. On one hand, HADS questionnaire evaluates anxiety and depression impact of tinnitus^16^. On the other hand, THI questionnaire measures the degree of tinnitus perception; emotional, physical, and social response to tinnitus; and tinnitus intrusiveness on audition^17^. Further details concerning the questionnaire-based monitoring of this study protocol can be found in^18^.

*EEG information* gives an insight into neuroplastic changes (if any) of acoustic therapies. The available EEG datasets make possible to undertake signal analysis in three modes: spontaneous, evoked, and induced EEG activity. In most of previous studies, only spontaneous activity has been analyzed, namely, participants were either at resting state or at listening to their acoustic therapy. However, EEG signals provide more in-depth neural information when they are related to specific motor, emotional, sensory, perceptual and/or cognitive events. This refers to evoked and induced EEG activity. Traditional patterns for evoked and induced EEG activity are event-related potentials and event-related (de-) synchronization, what can be estimated from the present databank. Spontaneous activity analysis helps to evaluate nature of ongoing activity. In contrast, evoke and induced activity analysis allows studying brain responses when individuals perceive the sound, pay attention to the stimulus, make a decision, and act accordingly. In brief, both bottom-up and top-down mechanisms associated with neuroplastic changes due to acoustic rehabilitation can be studied by using the present databank.

The present database was created by following a protocol formerly approved by the Ethical Committee of the National School of Medicine of the Tecnologico de Monterrey, described and published in^19^, and registered under the trial number: ISRCTN14553550. The database is available at^20^.

The study protocol was conducted as follows. All of the participants were informed about the experimental procedure and signed a consent form, where they authorized the publication of their collected data and the results of their follow-ups. Most of patients were recruited from the National Rehabilitation Institute, and they were additionally notified that their head physician was also following-up the investigation. Head physicians provided clinical histories and demographic information of patients.

All the patients who accepted to participate in the study were assigned to one of five groups: (1) TRT, (2) EAE, (3) ADT, (4) BBT and (5) placebo. Patients with no severe HL were randomly assigned to placebo and EAE groups. On the other hand, patients with a well-identified tinnitus pitch were included in TRT or ADT groups. Patients with tinnitus lower that 1 kHz were assigned to BBT. Control group (healthy volunteers) was asked to use the same relaxing music as placebo group. On average, 17 volunteers were assigned per group (see Table 1). Note that every acoustic therapy was adjusted to the HL level, tinnitus frequency and tinnitus intensity, expect for the relaxing music that was used as placebo.

**Fig. 2:**
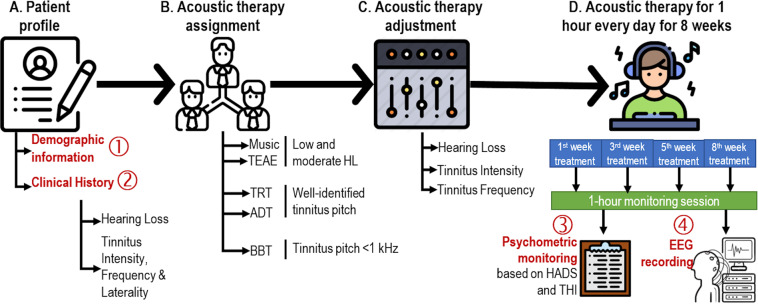
Study design. The present database contains (1) demographic information, (2) clinical history, and (3) psychometric and (4) EEG monitoring of 103 volunteers, of which 89 of them suffered from tinnitus, and the rest of them were controls.

All of the participants (both with and without tinnitus) were instructed to use the corresponding acoustic therapy for one hour every day, at any time of the day, and for eight weeks. The therapy was monitored four times along the two months: the first (session 1), the third (session 2), the fifth (session 3), and the eighth (session 4) week after having initiated the therapy. At each monitoring session, the THI and HADS questionnaires were applied, and an EEG recording was made, as illustrated in Fig. 2. It can be seen from Fig. 1 that only 71 of the 103 participants completed the experimental procedure. Although the repository has 103 files, not all of them are complete. This information is specified in the Excel file found in^20^.

## Methods

### Device for acoustic therapy

Audio players were provided for patients. These audio players had a 4 Gb memory, and included a pair of headphones, and a battery charger. The frequency response of headphones was tested to guarantee the proper reproduction of acoustic therapies, and volume limit was adjusted to the patient audiometry. However, some patients preferred to use their own devices. To record the daytime and the daily duration of acoustic therapies, they are currently offered in an online mode^21^.

### EEG data adquisition

**Fig. 3:**
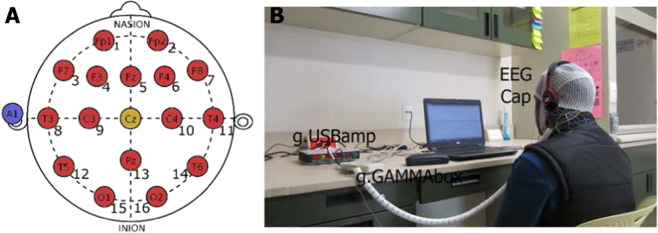
(**A**) *Electrode montage for EEG monitoring*. Two pre-frontal (FP1, FP2), five frontal (F7, F3, Fz, F4, F8), four temporal (T3, T4, T5, T6), two central (C3, C4), one parietal (Pz) and two occipital (O1, O2) recording sites are provided in the present database. Reference (blue) and ground (yellow) electrodes are not present in the EEG recording. (**B**) *g.tec system for EEG recording*. The g.tec system consisted of an electrode cap of medium size, a biosignal amplifier (g.USBamp), and an electrical isolation system for electrode power supply (g.GAMMAbox).

To record EEG activity, a *g.USBamp* amplifier of EEG signals was used. This system is a high-performance and high-accuracy biosignal amplifier with sixteen EEG channels (Fp1, Fp2, F7, F3, Fz, F4, F8, T3, C3, C4, T4, T5, Pz, T6, O1, and O2). These channels can be acquired up to 32 700 samples per second within a bandwidth between 0 and 100 Hz. For this study, the amplifier was configured to record the EEG signals at a sampling frequency of 256 Hz. The Cz channel was used to reference the rest of the channels, and left lobe ear worked as ground. The montage of the EEG channels was according to the 10/20 International System and is depicted in Fig. 3a. EEG electrodes were connected to the amplifier via the g.GAMMAbox, which refers to a battery system for electrode power supply, and patient electrical isolation. See Fig. 3b.

The *g.USBamp* was connected to the computer via OpenViBE platform, where experimental paradigm was implemented. OpenViBE was configured to digitally filter EEG signals by a band-pass, and a notch filters. The band-pass filter was a Butterworth design of 8^th^ order with cut-off frequencies between 0.1 and 100 Hz. The notch filter was similarly a Butterworth design of 4^th^ order at 60 Hz. Before each recording, OpenViBE was used to measure electrode impedance, which was equal to or lower than 5kohms for being acceptable.

### Experimental procedure for psychometric monitoring and EEG recording

Experimental procedure was undertaken in a well-equipped research laboratory with sufficient conditions to attend volunteers, and record EEG data. Laboratory conditions refer to a quiet atmosphere, water and toilet services, washing facilities, quiet air conditioner and parking place. The laboratory had a low background noise around 35 dBA.

**Fig. 4:**
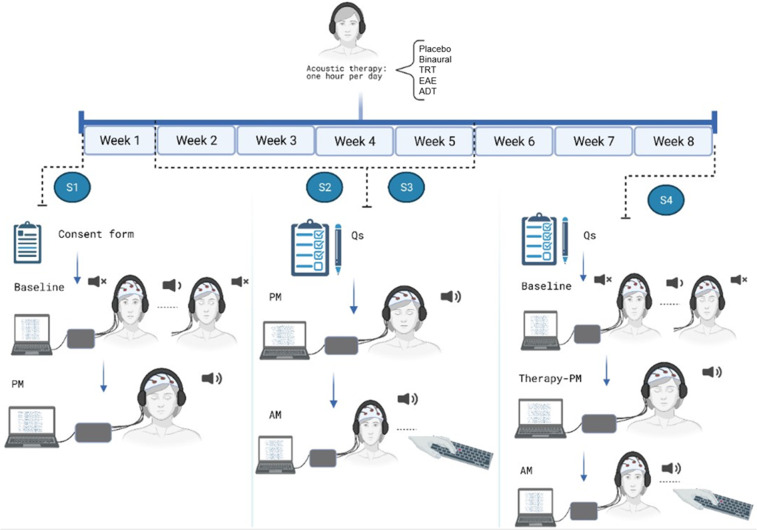
Experimental procedure. Acoustic therapies were monitored for eight weeks, along with four follow-ups. THI and HADS questionnaires were completed in S2, S3 and S4. EEG signals at resting state (baseline) for six minutes were recorded in S1 and S4. In addition, three minutes of EEG recording was undertaken in S4 when participants were listening to their corresponding acoustic therapy. PM for 2.5 minutes was recorded in all of the four sessions, and AM was recorded from S2 to S4.

As was mentioned above, acoustic therapies were applied for eight weeks, and monitored four times. THI and HADS questionnaires were completed in session 2 (S2), session 3 (S3) and session 4 (S4). EEG signals at resting state (baseline) for six minutes were recorded in session 1 (S1) and S4. In addition, three minutes of EEG recording was undertaken in S4 when participants were listening to their corresponding acoustic therapy. Finally, evoked/induced activity was recorded as well in two different modes: passive and active. Passive mode (PM) for 2.5 minutes was recorded in all of the four sessions, and active mode (AM) was recorded from S2 to S4. EEG recording procedure is described in detail below and illustrated in Fig. 4.

#### Baseline

Baseline was recorded in S1 and S4. The participants were asked to sit down on a comfort chair with their eyes open at looking at a fixed point on a computer screen for three minutes. After this three-min period, they listened a beep sound once, and they had been previously instructed to close their eyes for three minutes. In total, six minutes for baseline were registered.

#### Therapy

This mode was recorded at S4, when the assigned acoustic therapy had been already applied for eight weeks. For this mode, participants sat down with their eyes close (EC) and listened to their corresponding acoustic therapy for three minutes.

#### Passive mode (PM)

PM was the only condition recorded in the four sessions. Participants were stimulated around two minutes with a sequence of 50 auditory stimuli of one second with an inter-stimulus interval of two seconds. Participants were asked to keep their eyes closed during the auditory stimulation. Every auditory stimulus was in line with the acoustic therapy in use.

#### Active mode (AM)

AM was recorded at S2, S3, and S4, and EC was requested as possible. Three different soundscapes were played, while five associated auditory stimuli were randomly played. In order to identify auditory stimuli, participants pressed a keyboard button. The soundscapes, and their related auditory stimuli to be identified for each monitoring session were: (1) *restaurant*: (i) human sound (tasting food), (ii) microwave sound, (iii) glass breaking, (iv) door closing, and (v) soda can being opened; (2) *park*: (i) camera clicking, (ii) turning a book page, (iii) cards shuffling, (iv) human laughing, and (v) human whistling; and (3) *construction in progress*: (i) human sound (yelling), (ii) police siren, (iii) mobile dialing, (iv) bang and (v) hit. All the stimuli lasted 1 s and were repeated 50 times at a random rate.

### Processing

Raw signals are provided in the present database, in two formats: GDF and SET.

## Data Records

### Repository

This database was published on September 17^th^, 2021 at Mendeley Data^20^.

### Data files

On one hand, demographic, clinical and psychometric data of participants is provided in an Excel-file. This file must be read from left to right, and the top of the columns as headers. Each row represents a participant. In some cases, the information would not be available, and unavailability was represented as ‘- ’. EEG-files are provided in GDF and SET formats. Both of them can be imported by using the MATLAB open-source toolbox, EEGLAB.

### Data organization

**Fig. 5:**
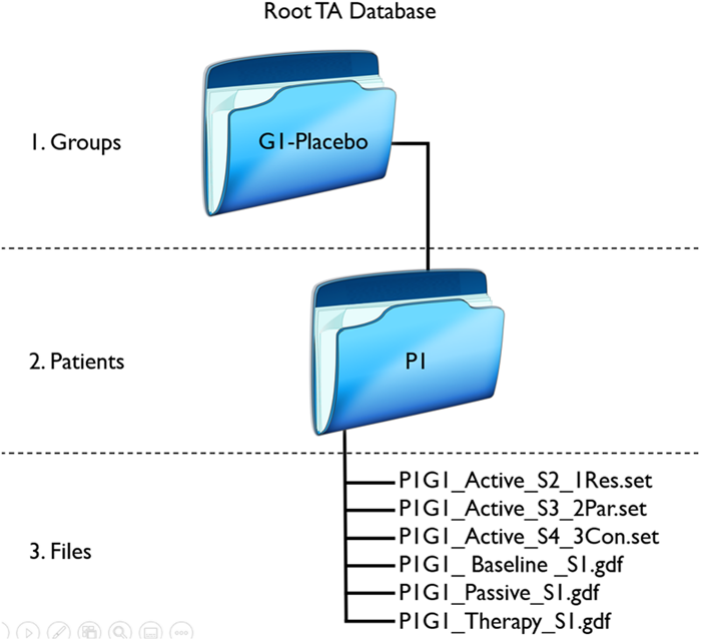
Representation of breakdown of the group, patients, and recording condition. All files are provided in GDF-format.

Excel-file and EEG-files are provided in the main folder. EEG-files are organized in three levels: (1) group, (2) patient, and (3) recording condition. See Fig. 5. Each patient folder contained six different conditions: (1) open- and close-eyes (‘OECE.gdf’), (2) PM (‘Passive.gdf’), (3) at listening therapy (‘Therapy.gdf’), (4) AM at restaurant soundscape (‘A1-Restaurant.gdf’), (5) AM at park soundscape (‘A2-Park.gdf’), and (6) AM at construction soundscape (‘A3-Construction.gdf’).

### EEG recording events

Interactions with and instructions given to participants during the EEG recording were marked with event numbers. In total, 13 events were marked along the GDF-files. Depending on the type of file, the events used in each experimental condition changed. Then, events per file type are defined in Table 2.

**Table 2:** Event codes.

EVENT	FILE TYPE			
	*OECE.gdf*	*Therapy.gdf*	*Passive.gdf*	*Active.gdf*
33024	Play ‘beep’ sound to indicate to close eyes	Play the acoustic therapy	Play one-second auditory stimulus	Release key P
33025	Stop ‘beep’ sound to indicate to close eyes	Stop the acoustic therapy	Stop one-second auditory stimulus	Play the soundscape at hand
33026				
33027				
33029				
33032				Press key i to start the session
33034				Press key P when identify one of the five sounds played randomly along the sound scape
33039	Recording completion	Recording completion	Recording completion	Recording completion
33041				Stop sound 1
33042				Stop sound 2
33043				Stop sound 3
33044				Stop sound 4
33045				Stop sound 5

Events refer to the instructions given to participants during the EEG recording, or the computer execution of multimedia material.

## Technical Validation

### Study design

**Fig. 6:**
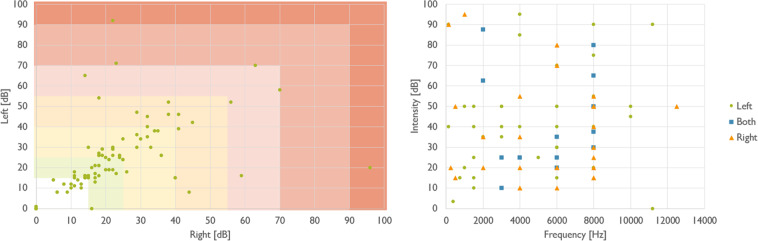
Hearing condition and tinnitus characteristics. On the left, HL level of participants in left (y-axis) and right (x-axis) ears is presented. The HL level is categorized in seven impairment levels: (1) no impairment (0–15 dB), (2) slight HL (15–25 dB), (3) mild HL (25–40 dB), (4) moderate HL (45–55 dB), (5) significant HL (55–70 dB), (6) severe HL (70–90 dB), and (7) profound HL (>90 dB). On the right, acoustic characteristics of the tinnitus in terms of intensity, frequency and laterality are presented.

A person-centred and needs-based approach that is a common practice in rehabilitation audiology was followed in the present study design. That is, each acoustic therapy was adjusted to the HL and acoustic tinnitus characteristics of each participant, what are indeed reported in Fig. 6. Furthermore, the selection of the therapy for each participant was not randomly allocated. The allocation was in line with HL level, and acoustic properties of tinnitus. For example, participants with low and moderate HL were allocated to placebo and EAE groups, those with a well-identified tinnitus pitched were treated with TRT and ADT groups, and those with a tinnitus frequency lower that 1 kHz were in BBT group. See Fig. 2.

On the other hand, only patients with subjective tinnitus were recruited. Tinnitus can be objective, when sound is generated in the body, or subjective, when no specific inner body sound source. To avoid recruiting patients with objective tinnitus, all of those with any history of otitis, cerebellopontine angle tumours, psychiatrist pathologies, demyelinating diseases of the nervous system, or epilepsy were excluded from the study protocol.

Finally, it is important to note that an age-matched control group, which followed the same procedure that experimental group, was included. The daily exposure to any kind of sound can lead to habituation not related to tinnitus palliation. Therefore, it was relevant to have a control group to monitor unrelated habituation changes.

### Methodological assessment

The present study was previously approved by the local Ethical Committee (CONBIOETICA19CEI00820130520), it was registered as a clinical trial (ISRCTN14553550) in BioMed Central (Springer Nature), the protocol was published in^19^, it attracted L’Oréal-UNESCO Organization as a sponsor, and six publications have resulted from the analysis of this database^5,9,10,18,19,22^.

### Sample tracking system

**Fig. 7:**
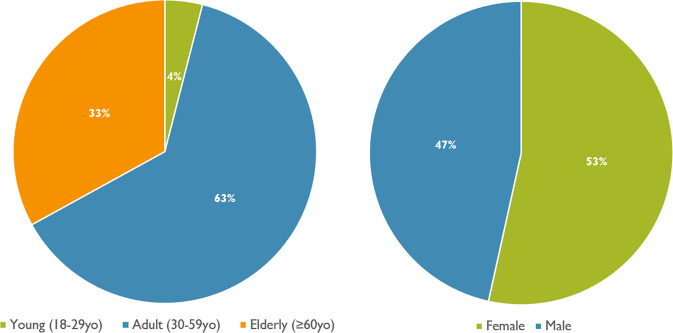
Demographic information of the sample. On the left, sample age in three categories (young, adult, elderly) is presented, where can be seen that most of participants were in adult age (30–59 years). On the right, sample gender is presented, where can be seen that majority of participants were females.

The sample tracking system adopted for this study was in four different directions: (1) a clinical support by the head-physician, (2) demographic information collection, (3) psychometric evaluation, and (4) EEG monitoring. Sample age and gender are presented in Fig. 7. Age was categorized in youth (18–29 years), adultness (30–59 years) and elderly (>60 years). As can be seen from the figure, most of participants were adults, and a well-balanced gender sample was recruited.

**Fig. 8:**
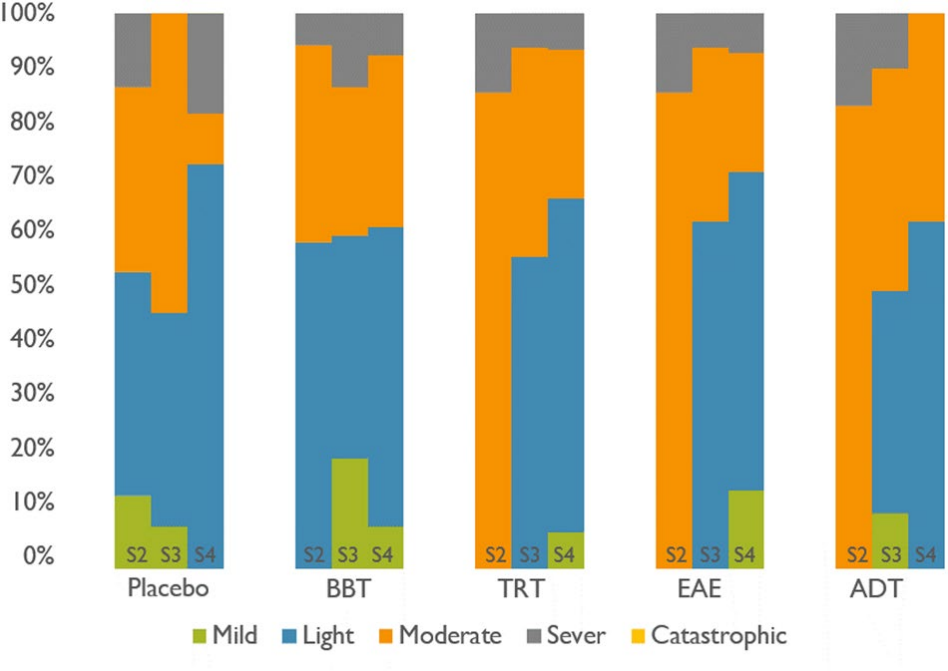
THI outcome for each group (placebo, BBT, TRT, EAE and ADT) in the three monitoring sessions (S2, S3 and S4). This questionnaire outcome is one of four conditions: light, mild, moderate, severe o catastrophic alteration.

**Fig. 9:**
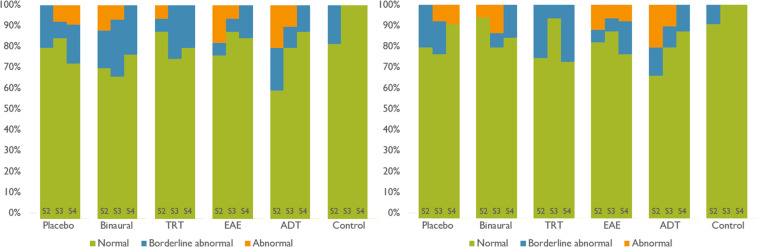
HADS outcome for each group (placebo, BBT, TRT, EAE and ADT) in the three monitoring sessions (S2, S3 and S4). This questionnaire outcome is one of three conditions: normal, abnormal and borderline abnormal. On the left, the stress-monitoring outcome is presented. On the right, the anxiety-monitoring outcome is shown.

In addition, a psychometric evaluation was undertaken during the acoustic treatment at three different stages of the procedure. This evaluation concerned tinnitus perception assessment (based on THI), and stress/anxiety monitoring (based on HADS). THI-evaluation revealed light, mild, moderate, severe or catastrophic impairment due to tinnitus along the acoustic treatment. See Fig. 8. Similarly, HADS-evaluation allowed detecting the changes of stress and anxiety due to tinnitus in three different ranges: normal, abnormal, and borderline abnormal. See Fig. 9.

Finally, an EEG-based monitoring throughout the acoustic therapies was carried out. EEG information is a window to understand the nature of tinnitus, and the possible neuroplastic changes provoked by acoustic therapies. EEG signals provided in this database can be analysed in three different modalities: (1) spontaneous, (2) evoked and (3) induced modality. From those modalities, neuro-markers can be estimated from spontaneous activity, and/or codification and retrieval of auditory material masked by tinnitus perception can be analysed from evoked and induced activity (among many other analyses).

**Fig. 10:**
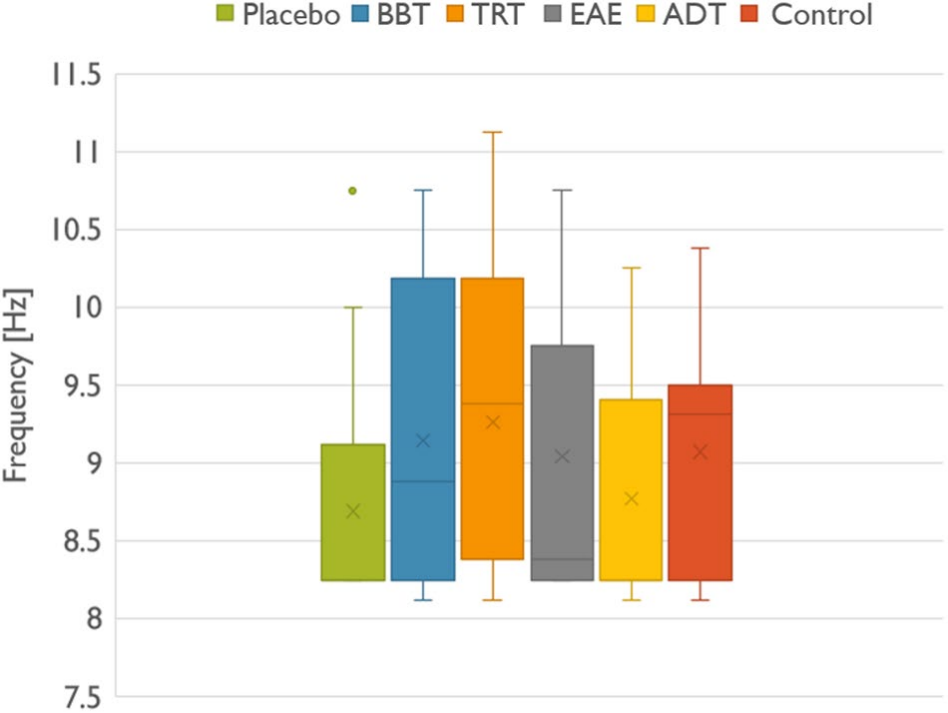
Individual alpha frequency of each volunteer participating in each experimental group. Placebo, binaural, TRT, EAE, ADT and control.

As a case in point, individual alpha frequency (IAF) was estimated from EEG signals in spontaneous modality (EO and EC condition), and shown in Fig. 10. Numerically, IAF refers to the peak value of the alpha band spectrum (8–13 Hz) at resting state over the occipital lobe^23^. IAF is around 10 Hz in healthy adult individuals, and reflects the functionality of the Central Nervous System, the mental health and the cognitive functions^24^. Several studies have demonstrated that IAF diminished when neuroplastic changes occurred in traumatic lesion, schizophrenia, attention deficit disorder, and post-traumatic stress. From the figure, it can be seen that mean IAF is below 10 Hz, as is expected owing to the neuroplastic changes provoked by refractory and chronic tinnitus. However, this result is not conclusive since control group has an IAF below 10 Hz, as the rest of the groups. This result is not surprising since IAF slowly decreases in elderly individuals, as well^25^. The main purpose of results in Fig. 10 is to show that EEG signals provided in this database are meaningful and technically valid for any research purpose pursued by the scientific community. Obviously, to obtain conclusive results, extensive analyses are necessary.

## Data Availability

The computer program to estimate the IAF can be found in^26^, and is described in^27^.
